# Editorial: Eliciting plant defense responses: From basic to applied science for sustainable agriculture

**DOI:** 10.3389/fpls.2023.1129305

**Published:** 2023-02-07

**Authors:** Artemio Mendoza-Mendoza, Mario Serrano, Kátia Regina Freitas Schwan-Estrada, Jorge F. S. Ferreira, José Abramo Marchese

**Affiliations:** ^1^ Faculty of Agriculture and Life Sciences, Department of Wine, Food and Molecular Biosciences, Lincoln University, Lincoln, New Zealand; ^2^ Centro de Ciencias Genómicas, Universidad Nacional Autónoma de México, Cuernavaca, Morelos, Mexico; ^3^ Alternative Control Plant Disease Lab., Agronomy Department, State University of Maringá, Maringá, Brazil; ^4^ US Salinity Laboratory (USDA-ARS), Riverside, CA, United States; ^5^ Plant Physiology and Biochemistry Lab., Agronomy Department, Federal Technological University of Paraná, Pato Branco, Brazil

**Keywords:** plant immunity, plant defense responses, elicitors, PAMPS, MAMPs

Plants constantly face a diversity of pathogens and insects that affect food production. Synthetic agrochemicals are often use to overcome these challenges. However, current demands for stringent worldwide regulatory policies led to the development of sustainable agriculture strategies, including naturally-derived molecules that elicit plant defense responses (Scariotto et al., 2021). The commercial use of these molecules is still limited, mostly due to poor knowledge on the molecular mechanisms producing their effects on plant metabolism. In recent decades, efforts have been directed toward understanding how individual molecules, such as immune receptors or microbial effectors, enable plants to perceive and respond to pathogens, insects, and other stresses. Furthermore, recent research on plant immunity has revealed high levels of complexity, including regulation mediated by micro-peptides and miRNA. Such knowledge opens the opportunity to link basic and applied science to facilitate using natural elicitors as a sustainable option for crop protection.

This Research Topic highlights emerging trends in plant defense response induced by elicitors, focusing on the progression from molecules to plant communities and crops, and interdisciplinary approaches to decipher the complexity of the mechanisms underlying plant-pathogen and plant-insect interactions.

Several publications herein bring important news about plant immunity. Klemm et al. sheds light on the evolution and regulation of a large protein family and provides a framework for a more detailed understanding of the molecular functions of Kiwellins. The biological function of Kiwellins is apparently linked to plant defense. Klemm et al. found that Kiwellins are evolutionarily conserved in various plant species and emerged in land plants (embryophyta) and are absent from fungi. They also introduce a systematic Kiwellin nomenclature based on a detailed evolutionary reconstruction of this protein family. Hwang et al. showed that an apoplastic effector Pat-1Cm of the gram-positive bacterium *Clavibacter michiganensis* acts as both a pathogenicity factor and an immunity elicitor in plants. The results indicate that Pat-1Cm is a distinct secreted protein carrying a functional catalytic triad for serine protease and this enzymatic activity might be critical for both pathogenicity and HR-eliciting activities of Pat-1Cm in plants.

Agisha et al. studied the sugarcane molecular defense mechanism in response to two *Sporisorium scitamineum* isolates with varying degrees of virulence to facilitate the development of smut-resistant sugarcane varieties. Functional analysis of differentially expressed genes (DEGs) revealed that most of them were associated with hormone signaling and the synthesis of defense-related metabolites, suggesting a complex network of defense mechanisms triggered in response to specific isolates of the smut pathogen. In turn, Zhang et al. explored how cucumbers can regulate photosynthesis, protective enzyme activity, and basic metabolism to resist fungal disease (*Sphaerotheca fuliginea*) and aphids (*Aphis gossypii*). The combination of powdery mildew infection and aphid infestation reduced photosynthesis and basal metabolism in cucumber plants, although the activities of several protective enzymes increased. The results showed that cucumber could enhance its pest/pathogen resistance by changing physiological metabolism when exposed to a complex infection system of pathogenic microorganisms and insects.

Wang et al. showed that the allotetraploid *Nicotiana benthamiana* recognizes two types of microbe-associated molecular patterns (MAMPs) by two homologous but diverged receptor-like proteins (RLPs), sugesting that an allopolyploid plant exhibits defense hybrid vigor by acquiring divergent immune receptors from different ancestor. Basu et al. introduced a novel therapy of Huanglongbing (HLB) and fire blight by enhancing the innate immunity of the host plant. Specifically, they constructed an *in silico* library of chimeras containing two different host peptides with observed or predicted antibacterial activity. Finally, they conducted *ex planta* studies to show that chimeras not only clear the causative bacteria from citrus leaves with HLB and from apple leaves with fire blight, but also augment host innate immunity during infection.

Plants rely on the perception of a multitude of herbivory-associated signals to activate their defenses to insect herbivores. These stimuli are mainly derived from three functional components, namely, mechanical damage, insect-associated microbe, and insect’s chemical cues (Schuman and Baldwin, 2016; Waterman et al., 2019). In this sense, the studies by Mao et al. broadened the understanding of how potato plants integrate responses to a multitude of stimuli upon herbivory by the potato tuber moth (*Phthorimaea operculella*, PTM) and evidenced that insect-associated microbes greatly modulated the plants response to insect herbivory. Dejana et al. using the tomato-*Funneliformis mosseae* mycorrhizal system, analyzed the effect of moderate differences in P fertilization on plant and pest performance, and on Mycorrhiza induced resistance (MIR) against biotic stressors including the fungal pathogen *Botrytis cinerea* and the insect herbivore *Spodoptera exigua*. The results showed that P influences mycorrhizal priming of plant defenses and the resulting induced-resistance is dependent on P availability, and suggest that mycorrhiza fine-tunes the plant growth *vs.* defense prioritization depending on P availability. Finally, their results highlight how MIR is context dependent, thus unraveling molecular mechanism based on plant defense that will contribute to improved efficacy of mycorrhizal inoculants for crop protection.

The editorial board of this special Research Topic - Eliciting Plant Defense Responses: from Basic to Applied Science for Sustainable Agriculture thanks the contributing authors for adding their knowledge to the field of plant immunity.

## Author contributions

JM, AM-M, KS-E, and MS contributed equally to the production of the Research Topic. All authors contributed to the production of the editorial.

## References

[B1] Scariotto S. Tomazeli V. N. Paladini M. V. De Oliveira Bolina C. Sobrinho R. L. Da Silva E. P.. (2021). Plant innate immunity in strawberry induced by pathogen-associated molecular pattern harpin and acibenzolar-s-methyl. Theor. Exp. Plant Physiol. 33, 357–367. doi: 10.1007/s40626-021-00218-w

[B2] Schuman M. C. Baldwin I. T. (2016). The layers of plant responses to insect herbivores. Annu. Rev. Entomol 61, 373–394. doi: 10.1146/annurev-ento-010715-023851 26651543

[B3] Waterman J. M. Cazzonelli C. I. Hartley S. E. Johnson S. N. (2019). Simulated herbivory: The key to disentangling plant defence responses. Trends Ecol. Evol. 34, 447–458. doi: 10.1016/j.tree.2019.01.008 30824196

